# Characteristics of Mpox Cases Diagnosed in Military Health System Beneficiaries, May 2022–April 2024

**Published:** 2024-09-20

**Authors:** Maura Metcalf-Kelly, Matthew Garrison, Ralph Stidham

**Affiliations:** 1 Integrated Biosurveillance Branch, Armed Forces Health Surveillance Division, Silver Spring, MD; 2 Human Health Services Directorate, U.S. Army Public Health Command-Pacific, Honolulu, HI; 3 Epidemiology and Disease Surveillance, U.S. Army Public Health Command-West, Joint Base San Antonio-Fort Sam Houston, TX

## Abstract

In May 2022, cases of mpox emerged beyond its historically endemic parts of Central and West Africa, primarily affecting men who have sex with men (MSM), and spreading via sexual networks. During this global outbreak the novel clinical and epidemiological characteristics of mpox disease were thoroughly documented in civilian populations but comparable data have not been reported for Military Health System (MHS) populations including beneficiaries. MHS cases were identified through a variety of data sources, including the Disease Reporting System internet (DRSi) and a customized query of ICD-10-CM (International Classification of Diseases, 10th Revision, Clinical Modification) encounter codes. Contemporaneous chart reviews of patients’ electronic health records in the Armed Forces Health Longitudinal Technology Application (AHLTA) and MHS GENESIS were performed to characterize cases. A total of 146 confirmed and probable MHS cases were identified from May 2022 through April 2024. Most cases occurred among MSM, with the majority developing classic prodromal symptoms and some experiencing anogenital and urinary symptoms.

**What are the new findings?:**

From May 2022 to April 2024, a total of 146 confirmed and probable mpox cases were identified among active duty service members and other MHS beneficiaries. With the majority of cases in MSM, most active duty service member cases were male (98.3%), 20-34 years old (81.4%), in the Army or Navy (39.8% each), and in the enlisted ranks (83.1%).

**What is the impact on readiness and force health protection?:**

Although historically atypical, the clinical and epidemiological characteristics of MHS mpox cases reflect those documented in civilian populations during the 2022 global outbreak. Department of Defense clinicians and public health officials should optimize public health messaging and education focusing on patient risk modification that target MSM.

## 1 BACKGROUND

In May 2022, while in the wake of the COVID-19 pandemic, the Department of Defense (DOD) and U.S. Government faced a new public health crisis: locally acquired cases of mpox (formerly monkeypox), a historically rare zoonotic disease, were emerging in multiple nonendemic countries and spreading among men who have sex with men (MSM) populations via sexual networks.^1^ While many of the initial cases and subsequent chains of transmission in Europe were linked to a LGBT+ Pride event on the Spanish island of Gran Canaria, by the end of May most cases were the result of local community transmission.^1^ By July 2022, over 16,000 cases had been reported from 75 mostly non-endemic countries including the U.S., prompting the World Health Organization (WHO) to declare mpox a Public Health Emergency of International Concern.^2^ Unusual characteristics of the virus during this outbreak—specifically, the extent of uninterrupted chains of human-to-human transmission, the form of spread, and population affected—reshaped a previously known disease into a new public health threat, with unclear impact to military readiness.

Mpox is a zoonotic disease caused by the monkeypox virus (MPXV), a double-stranded DNA virus of the *Orthopoxvirus* genus and *Poxviridae* family, which includes variola (smallpox), cowpox, and vaccinia viruses.^1^ Mpox was first detected in laboratory monkeys in 1958, and the first human case was discovered by WHO in 1970 in the Democratic Republic of Congo.^1^ WHO has declared mpox the most important *Orthopoxvirus* infection in humans since the eradication of smallpox.^3^

Two clades of MPXV exist: clade I (formerly the Congo Basin clade) causes more severe disease, with a potential case fatality rate of 10%; while clade II (formerly the West African clade), which includes subclades IIa and IIb, typically causes milder disease.^1^ The global mpox outbreak that began in 2022 has been driven by subclade IIb.^4^

Historically, human mpox cases were reported primarily in endemic areas of West and Central Africa, acquired via zoonotic transmission associated with hunting and preparing or consuming animal meat, or bites or scratches from infected primates and rodents.^1^ MPXV can also be transmitted among humans through direct contact with infectious sores or scabs, or through fomites contaminated with bodily fluids, as well as by respiratory secretion during prolonged close contact, and directly from an infected mother to her fetus.^1^ In the outbreak that started in 2022, however, the majority of cases have been linked to close intimate contact, particularly sexual activity. This outbreak has primarily affected MSM, who have presented with novel epidemiological and clinical characteristics.^4,5^

During MPXV’s 3- to 17-day incubation period an individual is not contagious. The consequent illness typically lasts 14-28 days.^5^ Prior to the 2022 outbreak, mpox illness usually began with prodromal symptoms including fever, malaise, headache, lymphadenopathy, and myalgias, followed by a rash that first appeared on the face and then spread over the body, to the palms of the hands and soles of the feet.^5^ Cases during the 2022 outbreak, however, were reported with atypical clinical presentations such as lesions first appearing in the anogenital region or mouth that did not always progress elsewhere on the body, along with rectal symptoms (e.g., pain with defecation, purulent or bloody stools), and prodromal symptoms that occurred after development of a rash—or did not occur at all.^4^ A person with mpox is contagious from the time of symptom onset until after all lesions have scabbed, sloughed from the body, with a fresh layer of renewed skin in place. Most infections with MPXV clade II, the lineage responsible for the outbreak of 2022, are mild to moderate in severity and self-limited, requiring only supportive care.^3,5,6^

Currently, 2 vaccines are licensed in the U.S. for the prevention of mpox, both of which are available through the Strategic National Stockpile: JYNNEOSTM (live, replication incompetent vaccinia virus) and ACAM2000® (live, replication competent vaccinia virus).^7^ JYNNEOS can also be used as post-exposure prophylaxis.^7^ The unique clinical and epidemiological picture of mpox patients in the 2022 outbreak have been well documented in civilian populations, both in the U.S and internationally.^8,9^ This report describes the characteristics of mpox cases identified in U.S. military personnel and other MHS beneficiaries.

## 2 METHODS

The surveillance period for this study was May 1, 2022 through April 30, 2024. The surveillance population included all active duty service members (including National Guard and Reserves) and other MHS beneficiaries seen at military hospitals and clinics during the surveillance period, including virtual consultations. Cases assessed in this report met the criteria for confirmed and probable mpox cases according to the U.S. Centers for Disease Control and Prevention (CDC)’s 2022 case definitions.^10^ A probable case must meet specific criteria: 1) no suspicion of other recent *Orthopoxvirus* exposure (e.g., *vaccinia virus* in ACAM2000 vaccination) and 2) demonstration of either the presence of *Orthopoxvirus* DNA by PCR of a clinical specimen; *Orthopoxvirus* using immunohistochemical or electron microscopy testing methods; or detectable levels of anti-orthopoxvirus IgM antibody between 4 to 56 days after rash onset. A confirmed case must meet 1 of 2 criteria: demonstration of the presence of MPXV DNA by PCR testing or Next Generation sequencing of a clinical specimen; or isolation of MPXV in culture from a clinical specimen.

Epidemiologists at the Armed Forces Health Surveillance Division (AFHSD)’s Integrated Biosurveillance (IB) Branch began tracking confirmed, probable, and potential mpox cases among MHS beneficiaries at the start of the global outbreak in May 2022. Mpox diagnoses were ruled out due to inconclusive, equivocal, or negative laboratory results, or insufficient information. IB epidemiologists used the DOD Electronic Surveillance System for the Early Notification of Community-based Epidemics (ESSENCE) to construct an mpox query using chief complaint keywords and discharge diagnosis codes from the International Classification of Diseases, 10th Revision, Clinical Modification (ICD-10-CM).^11^ The Electronic Data Interchange Personal Identifiers (EDI-PIs) associated with encounters flagged by this query were used to conduct chart reviews of potential cases’ electronic health records in the Armed Forces Health Longitudinal Technology Application (AHLTA) and MHS GENESIS.

AFHSD-IB created a master case list for mpox from various sources including the ESSENCE mpox query, DOD Reportable Medical Events (RMEs) entered in the Disease Reporting System internet (DRSi), service-specific reporting to AFHSD, Commanders’ and Directors’ Critical Information Requirements (CCIRs and DCIRs, respectively), the National Guard Bureau, and direct communications with other DOD partners. Cases were validated and populated with demographic, epidemiologic, and clinical data by conducting chart reviews of patients’ electronic health records, with key measures including sexual behavior, clinical signs and symptoms, duration of illness, and likely mode of transmission.

One reviewer with a background in epidemiology and health surveillance conducted chart reviews of all confirmed and probable MHS beneficiary cases that occurred from May 1, 2022 through April 30, 2024 for which electronic health records were available. Each patient’s records were reviewed from the date of the initial mpox-related health encounter through documented physician-authorized release from isolation or new in-person encounters for unrelated medical services.

Case locations were defined by the country (and U.S. state, where applicable) of the hospital or clinic where the patient was initially diagnosed. Signs and symptoms that occurred at any point in the patient’s course of illness were defined by the parameters established for chart review in the preceding paragraph. Patients were identified as MSM if their encounter notes explicitly established their MSM status, or alluded to a male spouse or partner or sexual encounter(s) with a male partner. The likely source or cause of mpox exposure was loosely grouped into the following categories: sexual/intimate, other person-to-person, animal, fomites, and unknown; with the determination made based on details shared in provider notes. Coinfections were defined by the presence of positive laboratory results for other communicable diseases—or provider documentation of such results—that were dated within a 1-week window of specimen collection or return of positive results for non-variola orthopoxvirus. Duration of illness was determined by calculating the number of days that elapsed from symptom onset to release from isolation.

## 3 RESULTS

**Figure 1:**
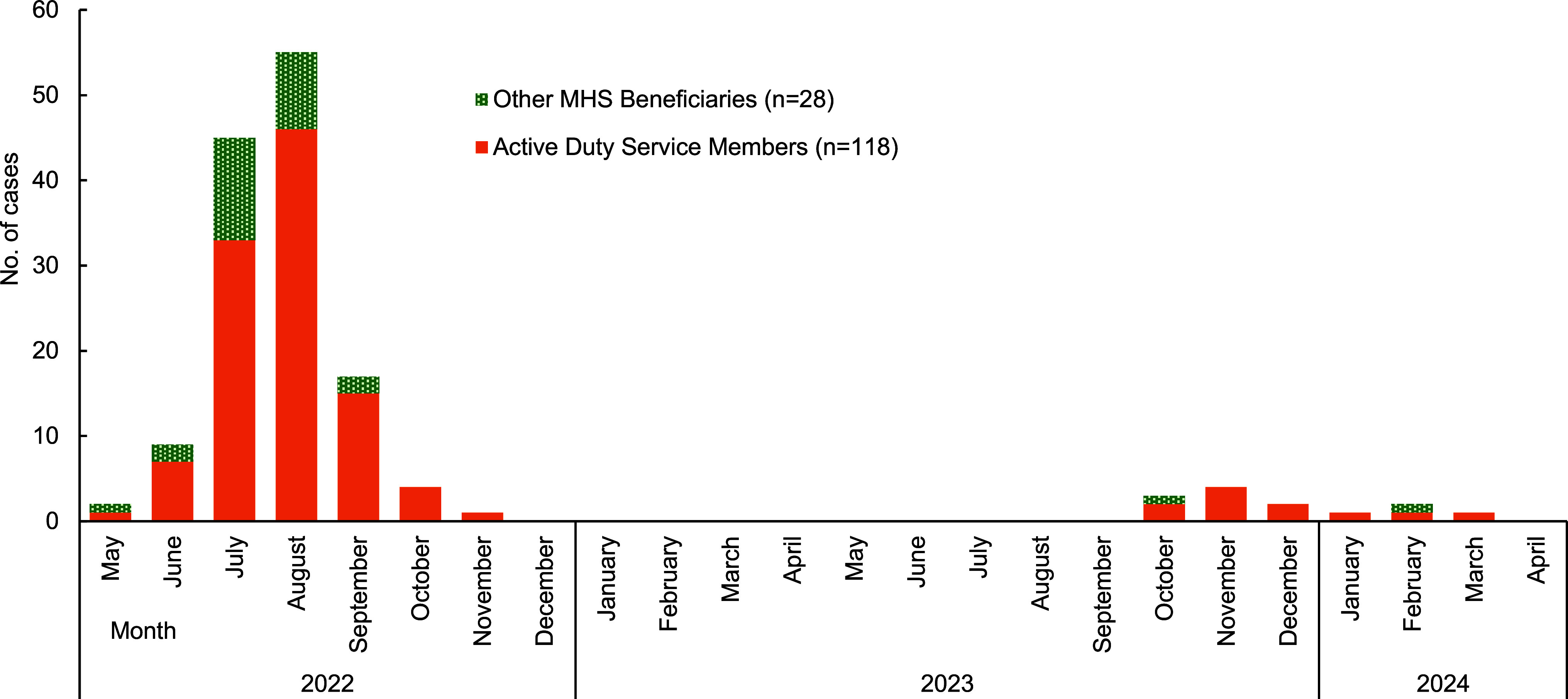
Confirmed and Probable Mpox Cases Among MHS Beneficiaries, by Month of Symptom Onset, May 2022–April 2024

A total of 146 confirmed and probable cases of mpox were identified among MHS beneficiaries during the study period. Symptom onset dates ranged from May 25, 2022 to March 9, 2024. After peaking in August 2022, with 55 cases, incidence among MHS beneficiaries declined sharply to 17 cases in September and then 4 cases in October, and has remained substantially lower since, with 2 or fewer cases per month from that time (**Figure 1**). This trend reflects trends seen in the U.S. civilian population.^6^ Clade-specific testing was conducted for at least 15 MHS beneficiary cases from Germany (n=2), Italy (n=2), Spain (n=2), the United Kingdom (n=1), and the U.S. (n=7), with all 15 identified as MPXV clade II (data not shown).

**Figure 2:**
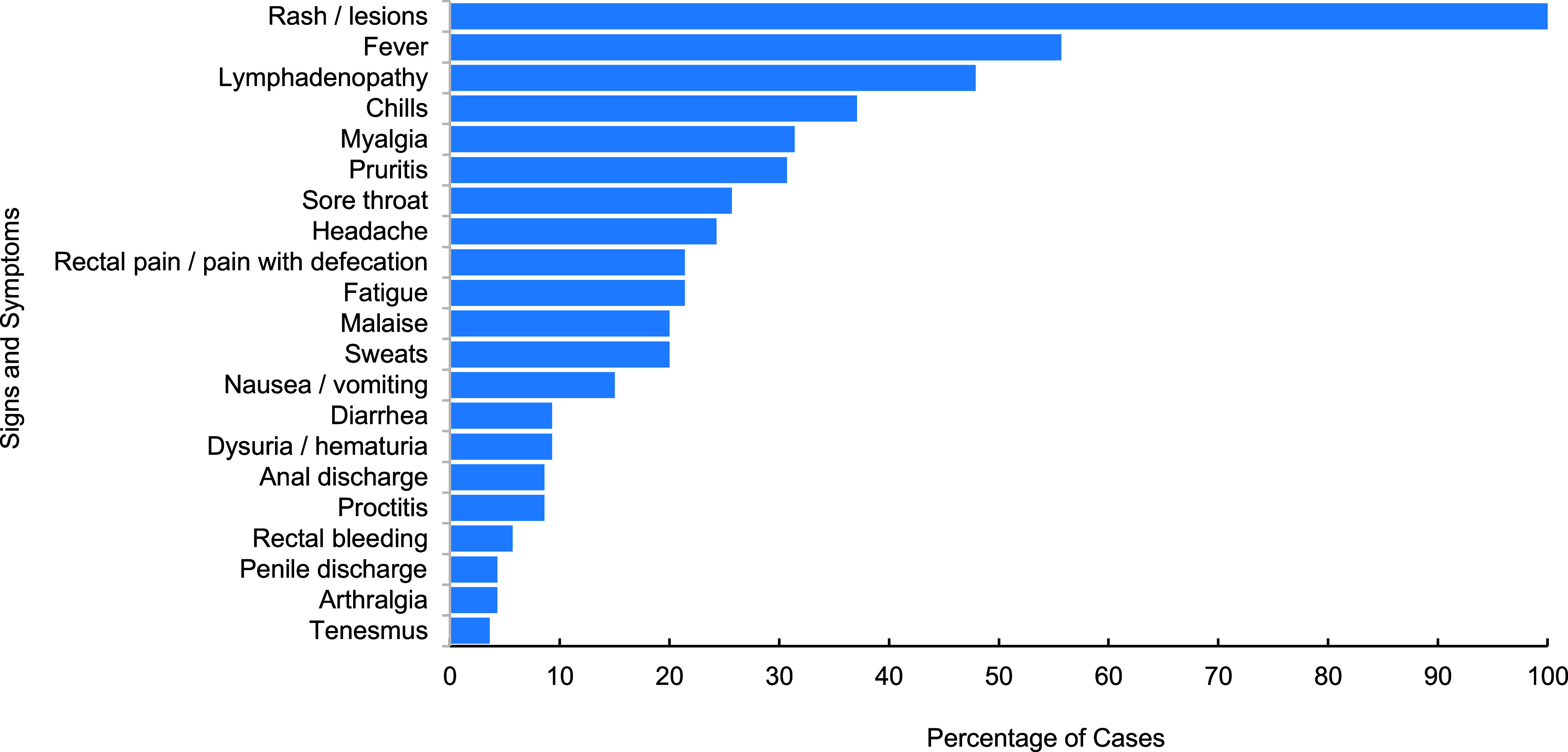
Sign and Symptom Frequency Among MHS Beneficiary Mpox Cases, May 2022–April 2024

Active duty service members accounted for the majority (80.8%) of cases, with other MHS beneficiaries, including retirees, accounting for 19.2% (**Table**). The majority (89.0%) of active duty service member cases were diagnosed in the U.S., with California, Virginia, and Maryland collectively accounting for more than half of these cases (**Table**). Most active duty service member cases were male (98.3%), 20-34 years old (81.4%), in the enlisted ranks (83.1%), in the Army or Navy (39.8% each), and either non-Hispanic Black (33.9%) or non-Hispanic White (29.7%) race or ethnicity (**Table**). Among cases with confirmed symptom onset dates and quarantine release dates (n=93, active duty service members; n=19, other MHS beneficiaries), the average time from onset to resolution and release from isolation was 29 days (data not shown). All MHS beneficiary patients experienced a rash, over half experienced a fever (55.7%), and nearly half (47.9%) had lymphadenopathy (**Figure 2**).

Among patients with information about sexual behavior (n=109, active duty service members; n=27, other MHS beneficiaries), 88.8% and 100% of cases, respectively, occurred in MSM (data not shown). At least 30 (20.5%) of all MHS beneficiary cases were coinfected with 1 or more communicable diseases, including 24 patients (16.4%) who tested positive for a sexually transmitted infection (STI) (data not shown). Among MHS beneficiaries for whom a likely source or cause of disease exposure could be determined (n=37), the majority (81.2%) were attributable to sexual or intimate activity; other person-to-person contact accounted for 18.3% of cases, with transmission via fomites identified as a possible source of exposure for 4.6% of these cases (data not shown).

## 4 DISCUSSION

The characteristics of MHS mpox cases described in this report provide a foundation for additional studies to enable public health entities and military leadership alike to stratify at-risk population demographics, anticipate impacts on military readiness, and develop appropriate and effective mitigation strategies. These data also provide clinicians with an additional tool to assist diagnosis and treatment, most likely clinical presentation when mpox is a differential diagnosis, and populations at risk.

Active duty service members ages 25-29 years demonstrated the highest mpox occurrence of any single age group, and at least 83.1% of active duty cases were in the enlisted force. The overall incidence rate among active component personnel (approximately 1.6 million people) was 6.7 cases per 100,000 persons, which is lower than the rate of 9.9 cases per 100,000 in the U.S. civilian population (about 333 million people) during the same time period.^12,13^ In relation to active component populations (which exclude activated National Guard and Reserves) during the study period, Defense Medical Surveillance System data show that the Navy (population 402,920) had the highest cumulative incidence percentages of mpox cases, at 1.2%, followed by the Army (pop. 568,520) at 0.7%, the Air Force (pop. 377,804) at 0.4%, and the Marine Corps (pop. 227,407), at 0.4%. Based on these data, the impact of this disease on military readiness appears to be minimal, at least in relation to the clade that drove global transmission during the surveillance period.

Clinical presentation of mpox in MHS beneficiaries mirrored what has been documented in affected civilian populations during the global outbreak.^4,8^ Many cases developed classic prodromal symptoms including fever, lymphadenopathy, sore throat, fatigue, and malaise. Some cases, however, experienced anogenital and urinary symptoms that were atypical prior to 2022 but have been frequently documented in civilian populations since 2022, including rectal pain or pain with defecation, diarrhea, proctitis, anal discharge, rectal bleeding, and tenesmus. Average duration of illness and associated isolation among all MHS beneficiaries was 29 days. While a comparable metric could not be found for the U.S. civilian population, CDC guidance indicates that mpox illness typically lasts 2-4 weeks.^5^

Approximately 88.8% of active duty service member cases occurred in MSM, which is slightly lower than in the overall U.S. population (90%).^6,12^ At least 20.5% of all MHS beneficiary cases were coinfected with 1 or more communicable diseases, including 16.4% testing positive for an STI. The majority of MHS cases for which a likely source or cause of disease exposure could be determined were attributable to sexual or intimate activity. This finding suggests that DOD health care providers and public health entities should optimize public health messaging that targets these groups, and may usefully adapt some of the tools already developed in the U.S. civilian sector for this purpose.

A case series that specifically describes mpox disease progression among MHS beneficiary cases, including details on when, and if, classic prodromal symptoms appeared, and location and progression of bodily lesions, would be valuable source of additional information. Such clinical information, when integrated with data presented herein on case symptomatology as well as demographic and epidemiological case characteristics, could support MHS clinicians’ identification of potential mpox cases in MHS patients, along with the incorporation of mpox into differential diagnoses where appropriate. DOD clinicians should also standardize consistent patient risk modifying education for this population, including vaccination recommendations, particularly in clinical settings where beneficiaries are seeking testing for STIs or other sexual health concerns.

Future discussion and study about the factors influencing the sharp decline in mpox incidence within the MHS beneficiary population is also warranted, to confirm, contradict, or modify assumptions informed by emerging evidence. Global civilian studies have concluded that mpox cases in the ongoing outbreak have been predominately confined to a specific patient population, i.e., MSM with multiple sexual partners.^6,9^ Consequently, it has been posited that effective targeting of pre- and post-exposure vaccine prophylaxis campaigns towards the MSM population along with decreased high-risk sexual activities due to awareness campaigns by DHA personnel may have contributed to the significant decline in MHS beneficiary cases.

## Figures and Tables

**Table:** Demographic and Military Characteristics of Confirmed and Probable Mpox Cases Among MHS Beneficiaries, May 2022–April 2024

	Beneficiary Type					
	All MHS Beneficiaries		Active Duty Service Members^a^		Other MHS Beneficiaries^b^	
	No.	%	No.	%	No.	%
Total	146	--	118	--	28	--
Branch of service						
Army	61	41.8	47	39.8	14	50.0
Navy	54	37.0	47	39.8	7	25.0
Air Force	22	15.1	15	12.7	7	25.0
Marine Corps	8	5.5	8	6.8	--	--
U.S. Public Health Service	1	0.7	1	0.9	--	--
Sex						
Male	143	97.9	116	98.3	27	96.4
Female	3	2.1	2	1.7	1	3.6
Race and ethnicity						
Black, non-Hispanic	45	30.8	40	33.9	5	17.9
White, non-Hispanic	42	28.8	35	29.7	7	25.0
Hispanic^c^	21	14.4	17	14.4	4	14.3
Asian/Pacific Islander	7	4.8	7	5.9	--	--
Other/unknown^d^	31	21.2	19	16.1	12	42.9
Rank						
E1-E4 (Junior enlisted)	--	--	55	46.6	--	--
E5-E9 (Senior enlisted)	--	--	43	36.4	--	--
O1-O3; W1-W3 (Junior officer)	--	--	11	9.3	--	--
O4-O10; W4-W5 (Senior officer)	--	--	4	3.4	--	--
Other/unknown^e^	--	--	5	4.2	--	--
Age group, y						
<20	1	0.7	1	0.9	--	--
20–24	27	18.5	24	20.3	3	10.7
25–29	50	34.2	44	37.3	6	21.4
30–34	32	21.9	28	23.7	4	14.3
35–39	17	11.6	12	10.2	5	17.9
40–44	8	5.5	5	4.2	3	10.7
45–49	4	2.7	3	2.5	1	3.6
>=50	6	4.1	1	0.9	5	17.9
Unknown	1	0.7	--	--	1	3.6
Location of diagnosis^f^						
Germany	5	3.4	5	4.2	--	--
Italy	5	3.4	4	3.4	1	3.6
Japan	1	0.7	--	--	1	3.6
Spain	4	2.7	3	2.5	1	3.6
United Kingdom	1	0.7	1	0.8	--	--
**United States**	**130**	89.0	**105**	89.0	**25**	89.3
Virginia	24		19		5	
California	23		21		2	
Maryland	18		13		5	
Florida	10		9		1	
Texas	9		7		2	
Washington	8		6		2	
North Carolina	7		4		3	
South Carolina	5		4		1	
Georgia	4		4		--	
Colorado	4		3		1	
Hawaii	3		2		1	
New York	2		1		1	
Illinois	2		1		1	
Alabama	1		1		--	
Arizona	1		1		--	
Indiana	1		1		--	
Kentucky	1		1		--	
Louisiana	1		1		--	
Massachusetts	1		1		--	
Missouri	1		1		--	
Oklahoma	1		1		--	
Pennsylvania	1		1		--	
Unknown	1		1		--	

Abbreviations: MHS, Military Health System; No., number; y, years.

^a^Includes National Guard and Reserves.

^b^Includes retirees.

^c^Includes White (n=12), Black (n=3), Other race (n=5), and Unknown race (n=1).

^d^Includes Other race/non-Hispanic (n=9), White/unknown ethnicity (n=5), Unknown race/ethnicity (n=10), Black/unknown ethnicity (n=4), Asian or Pacific Islander/Hispanic (n=2), and Other race/unknown ethnicity (n=1).

^e^Includes 1 cadet.

^f^Refers to location of health care facility where case was diagnosed.

## AUTHOR AFFILIATIONS

Integrated Biosurveillance Branch, Armed Forces Health Surveillance Division, Silver Spring, MD: Ms. Metcalf-Kelly; Human Health Services Directorate, U.S. Army Public Health Command-Pacific, Honolulu, HI: COL Garrison; Epidemiology and Disease Surveillance, U.S. Army Public Health Command-West, Joint Base San Antonio-Fort Sam Houston, TX: Dr. Stidham
